# Variety ACEs and risk of developing anxiety, depression, or anxiety-depression co-morbidity: the 2006–2022 UK Biobank data

**DOI:** 10.3389/fpsyt.2023.1233981

**Published:** 2023-12-27

**Authors:** Peilin Yu, Zhou Jiang, Chu Zheng, Ping Zeng, Lihong Huang, Yingliang Jin, Ke Wang

**Affiliations:** ^1^Department of Biostatistics, School of Public Health, Xuzhou Medical University, Xuzhou, Jiangsu, China; ^2^Key Laboratory of Human Genetics and Environmental Medicine, Xuzhou Medical University, Xuzhou, Jiangsu, China; ^3^Key Lab of Environment and Health, Xuzhou Medical University, Xuzhou, Jiangsu, China; ^4^Center for Medical Statistics and Data Analysis, Xuzhou Medical University, Xuzhou, Jiangsu, China; ^5^Jiangsu Engineering Research Center of Biological Data Mining and Healthcare Transformation, Xuzhou Medical University, Xuzhou, Jiangsu, China; ^6^Department of Biostatistics, Zhongshan Hospital, Fudan University, Shanghai, China

**Keywords:** ACEs, anxiety-depression co-morbidity, UK Biobank, restricted cubic spline, sex

## Abstract

**Objectives:**

Adverse childhood experiences (ACEs) and anxiety-depression co-morbidity are attracting widespread attention. Previous studies have shown the relationship between individual psychiatric disorders and ACEs. This study will analyze the correlation between anxiety-depression co-morbidity and different levels of ACEs.

**Methods:**

Seven categories of ACE and four classifications of psychiatric disorders were defined in a sample of 126,064 participants identified by the UK Biobank from 2006–2022, and correlations were investigated using logistic regression models. Then, to explore nonlinear relationships, restricted spline models were developed to examine differences in sex and age across cohorts (*n* = 126,064 for the full cohort and *n* = 121,934 for the European cohort). Finally, the impact of the category of ACEs on psychiatric disorders was examined.

**Results:**

After controlling for confounders, ACEs scores showed dose-dependent relationships with depression, anxiety, anxiety-depression co-morbidity, and at least one (any of the first three outcomes) in all models. ACEs with different scores were significantly positively correlated with the four psychiatric disorders classifications, with the highest odds of anxiety-depression co-morbidity (odds ratio [OR] = 4.87, 95% confidence intervals [CI]: 4.37 ~ 5.43), *p* = 6.08 × 10^−178^. In the restricted cubic spline models, the risk was relatively flat for females at ACEs = 0–1 and males at ACEs = 0–2/3 (except in males, where ACEs were associated with a lower risk of anxiety, all other psychiatric disorders had an increased risk of morbidity after risk smoothing). In addition, the risk of having anxiety, depression, anxiety-depression co-morbidity, and at least one of these disorders varies with each category of ACEs.

**Conclusion:**

The prevalence of anxiety-depression comorbidity was highest across ACE scores after controlling for confounding factors and had a significant effect on each category of ACEs.

## 1 Introduction

Originally, the definition of Adverse Childhood Experiences (ACEs) was limited to the experience of domestic abuse or the presence of family dysfunction during childhood (1); however, the definition has now been expanded to include other related factors such as community dysfunction and peer dysfunction (2), which can lead to many negative impacts on the organism in adulthood, such as psychiatric disorders and common chronic diseases (3). According to the Centers for Disease Control and Prevention (CDC), the local Child Protective Services (CPS) counted 686,000 children in the United States who were injured as a result of ACEs (3). Most previous studies have used the ACE questionnaire developed by Felitti to detect 3 categories of child maltreatment (including physical, verbal, and sexual abuse) and 4 categories of family dysfunction (exposure to substance abuse, psychiatric disorders, violent treatment of mother or stepmother, and criminal behavior in the home) in children under or equal to 18 years of age (1). Subsequently, studies have expanded the measurement of adversity at the social dimension (4) and the ACEs International Questionnaire (ACE-IQ) has included social factors (5). Over the past 20 years, research on ACEs has focused primarily on North America (6). However, because of its widespread damage to adult mental health, addiction, and life expectancy (7, 8), many other countries (including Europe) have begun to study ACE (9).

In recent years, the Epidemiological Research Center Depression Scale (CES-D) has been reported to have high detection rates in countries such as Italy (37%) and Spain (49%), which has prompted us to study psychiatric disorders in European countries (10). Early studies have found a strong association between depression and suicidal ideation (11, 12), with approximately 58% of patients with major depressive episodes reporting suicidal thoughts (13), which suggests that the serious risk posed by psychiatric disorders to an individual’s health cannot be ignored. Recent studies have shown that current evidence does not support the hypothesis that depression is caused by reduced serotonin activity or concentration (14). However, in the case of ACE, early adverse emotions associated with it alter normal psychological development, leading to psychologization (15) and mood disorders (16), triggering a biological stress response leading to effects on the hypothalamus-pituitary–adrenal (HPA) axis (17–19) that stimulate cortisol secretion from the adrenal cortex, which persists at high levels for long periods placing individuals at an increased risk for depression and anxiety. The development of psychiatric disorders may also be associated with the cumulative number of ACEs (18). Recent studies have found: that early ACEs worsen psychiatric problems in children (20) and the middle old-age (21); there is a dose-dependent relationship between ACEs and the development of chronic diseases or other risky behaviors in adulthood (22), also including psychiatric disorders (23). Of these, depression or anxiety disorders are the most common, and in some literature, it has been shown that the prevalence of depression is usually higher than that of anxiety (18, 19, 24). However, in the specific category of ACE, anxiety is more closely related to sexual or physical abuse, and depression is more closely related to emotional abuse (25).

Many previous studies have examined the relationship between ACEs and anxiety or depression, but to our knowledge, fewer studies focused on examining the relationship between anxiety-depression comorbidity (the co-occurrence of anxiety and depressive symptoms in individuals) (3, 19, 23, 26, 27). By the ICD-10 criteria, anxious depression not only requires the former condition to be met, but neither manifestation is the primary symptom. And if both manifestations can reasonably be diagnosed separately it cannot be said to be anxious depression (28). From reading the literature we have learned that, compared to non-anxious depression, anxious depression suffers from increased clinical symptoms, more frequent depressive episodes, more pronounced symptoms, and even an increased risk of suicide (28).

Therefore, the present study was designed to test the three hypotheses we proposed. First, we wanted to examine whether people who experience ACE are at increased risk for anxiety-depression co-morbidity, anxiety, depression, or at least one of these (any of the first three outcomes); and whether anxiety-depression co-morbidity is most strongly associated with ACE. In addition, we used restricted triple spline methods to assess the dose–response relationship between ACEs and psychiatric disorders in different sex groups and performed sensitivity analyses in European cohort. Also, we explored the dose–response relationship between ACEs and psychiatric disorders in different age groups. Finally, we examined the relationship between the categories of ACE and anxiety-depression comorbidity, anxiety, depression, or at least one of these (any of the first three outcomes).

## 2 Materials and methods

### 2.1 Data sources

This study used data from UK Biobank, an ongoing prospective population-based cohort study,^1^ the aim is to accurately and comprehensively assess environmental, psychosocial, genetic, and non-genetic factors related to exposure and outcomes and further analyze their relationships. From 2006 to 2010, 500,000 people aged 37–73 were recruited from 22 assessment centers in the UK, including touch screens, physical measures, and biological sampling (29).

**Figure 1:**
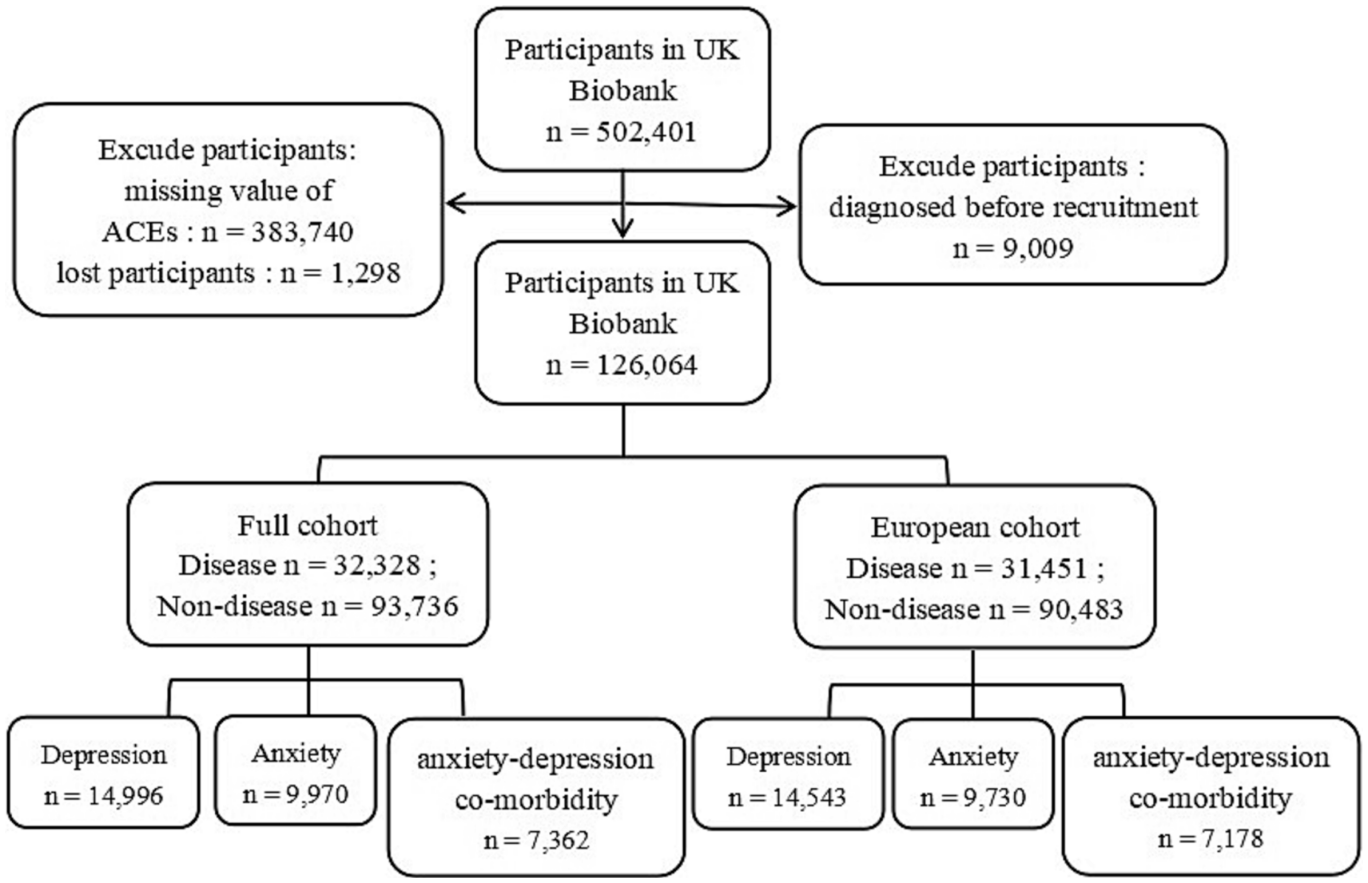
Flow chart for filtering participants in UK Biobank.

To investigate whether the categories of ACEs and different scores led to an increased risk of developing new cases of psychiatric disorders in the UK Biobank database, we established exclusion criteria for all participants: (1) those who had been lost to follow-up by 2022 for any reason, (2) participants lacking information on what was defined as an ACE, and (3) those who had a confirmed diagnosis of psychiatric disorders prior to recruitment, resulting in the inclusion of 126,064 participants (55,481 males and 70,583 females). The detailed process of participant selection is shown in Figure 1.

#### 2.1.1 Ascertainment of ACEs

First, the content of the Conflict Tactics Scale (CTS) (30) includes detailed questions about emotional abuse, physical abuse, and domestic violence; second, the Child Trauma Questionnaire (CTQ) (31) includes detailed questions that measure emotional and physical neglect, with some items being reverse-scored. Simultaneously, this is all relative to children under 18. We used an ACE questionnaire (32) based on the above questions, which then corresponded to the variables scale of the UK Biobank, while early life factors, family history reports, and traumatic events in online mental health questionnaires were assessed. Ultimately, we included seven types of ACEs in this study (felt hated by family members as a child, physically abused by family as a child, felt loved as a child, sexually molested as a child, someone to take to the doctor when needed as a child, maternal smoking around birth, and having a family history of psychosis). It’s worth noting that feeling loved as a child and someone to take to the doctor when needed as a child are reverse scores, which we re-coded for analysis (30, 31). The above ACEs were created as a binary variable (0 = no, 1 = yes). The seven types were then combined to calculate the total number of ACEs, yielding a range of 0–7 scores, which were divided into five groups (0, 1, 2, 3, ≥ 4) based on the total number in subsequent analysis (32). In addition, we describe the corresponding categories of ACEs in the ACE questionnaire in Supplementary Table S1.

#### 2.1.2 Ascertainment of outcome

The outcomes in this study were depression, anxiety, anxiety-depression co-morbidity, or at least one (any of the first three outcomes), and they were all defined as binary variables (0 = no illness, 1 = illness). We determined the number of participants based on admission data ICD-10 (main conditions of anxiety disorders, bipolar disorder, depression, and recurrent depression) (33, 34), and self-reported disease diagnoses (non-cancer disease codes) recorded in the database. Addresses for admission data and reasons for admission were obtained by linking to records from Health Event Statistics (England and Wales) and Scottish Morbidity Records (Scotland). Specific information can be found online.^2^ A record of the diagnosis of psychiatric disorders is provided in Supplementary Table S2. And anxiety-depression co-morbidity is defined as the simultaneous occurrence of anxiety and depressive diagnosis in an individual. It is important to note that the diagnoses of psychiatric disorders that we defined as new cases all occurred after the 2006–2010 recruitment through the July 19, 2022 cutoff.

#### 2.1.3 Ascertainment of confounders

Demographic information about the covariates of 126,064 participants in the UK Biobank from 2006–2022 was studied for age (years), sex (0 = female, 1 = male), smoking status (0 = never, 1 = previous, 2 = current), alcohol drinker status (0 = never, 1 = previous, 2 = current), education (1 = university degree, 2 = below university or other professional qualifications, 3 = none of the above), International Physical Activity Questionnaire (IPAQ) activity group (0 = low, 1 = medium, 2 = high), ethnic background (1 = European, 2 = European or Asian or African mixed race, 3 = Asian, 4 = African, 5 = others), BMI (1 ≤ 18.5, 2 = 18.5–24.9, 3 = 25–29.9, 4 ≥ 30), Townsend Deprivation Index (TDI) (35) at recruitment, which represents socioeconomic status. The above confounders are included in this study.

### 2.2 Statistical analysis

First, descriptive statistics were performed on the participants’ baseline characteristics and outcome variables. Continuous variables were expressed as means (standard deviation, [SD]), and categorical variables were expressed as frequencies (percentages). In addition, after stratification by ACEs scores, chi-square and Kruskal-Wallis H tests were used to check the significance of differences between participants. Logistic regression models were constructed to assess the relationship between ACE scores and depression, anxiety disorders, anxiety-depression co-morbidity, or at least one of these. Next, to test the robustness of the above relationships, we first tested the prevalence of ACE and psychiatric disorders across sex groups (*n* = 70,583 for females and *n* = 55,481 for males). Then, we constructed restricted cubic spline models (with 4 nodes at the 25th, 50th, 75th, and 95th quartiles) to test for differences in ACE in the full and European populations (36). Finally, we examined the correlation between the category of ACEs and each outcome variable. For missing values of variables, we performed multiple interpolations using the MICE package (37), and the proportion of missing data is described in Supplementary Table S3. The level of statistical significance was determined as 95% and bilateral (*p* < 0.05), and correlations were expressed as OR with 95% confidence intervals (CI). All the above analyses were performed in R software (R 2.4.1).

## 3 Results

### 3.1 Baseline comparison

The baseline characteristics are shown in Table 1. Overall, ACEs occurred in 55.59% of all participants aged 38–73. ACEs scores of 1, 2, 3, and ≥ 4 accounted for 33.79, 14.42, 5.37, and 2.01%, respectively. Also, all variables were associated with ACEs scores and all differences were significant (*p* < 0.001). Meanwhile, among all ACEs scores, females (55.99%) had more ACEs than males (44.01%) in the full cohort. Compared to other ethnic background groups, the number of Europeans is the highest (96.72%). Among other variables, most of the incidence of ACEs occurred in categories below college education (79.68%), history of alcohol consumption (94.83%), and overweight (41.31%). From the content of ACEs score and psychiatric disorders, it was clear that depression (24.64%), anxiety (10.21%), anxiety-depression co-morbidity (20.06%), and at least one (54.91%) accounted for the highest proportion when the ACEs score ≥ 4.

**Table 1:** The characteristics of the selected participants grading according to different ACEs scores in UK Biobank.

	Level	Overall	ACEs Score					*p* value
			Score 0	Score 1	Score 2	Score 3	Score 4	
*N* (%)		126,064	55,984 (44.41)	42,595 (33.79)	18,179 (14.42)	6,769 (5.37)	2,537 (2.01)	
Age (Mean ± SD)		55.852 (7.77)	56.36 (7.89)	55.93 (7.61)	55.05 (7.64)	54.16 (7.57)	53.72 (7.60)	<0.001
Sex (%)	Female	70,583 (55.99)	31,034 (55.43)	23,288 (54.67)	10,294 (56.63)	4,182 (61.78)	1,785 (70.36)	<0.001
	Male	55,481 (44.01)	24,950 (44.57)	19,307 (45.33)	7,885 (43.37)	2,587 (38.22)	752 (29.64)	
Smoking status (%)	Never	73,539 (58.33)	34,591 (61.79)	24,783 (58.18)	9,669 (53.19)	3,321 (49.06)	1,175 (46.31)	<0.001
	Previous	43,783 (34.73)	18,232 (32.57)	14,826 (34.81)	6,966 (38.32)	2,737 (40.43)	1,022 (40.28)	
	Current	8,742 (6.93)	3,161 (5.65)	2,986 (7.01)	1,544 (8.49)	711 (10.50)	340 (13.40)	
Alcohol drinker status (%)	Never	3,545 (2.81)	1740 (3.11)	1,120 (2.63)	427 (2.35)	173 (2.56)	85 (3.35)	<0.001
	Previous	2,968 (2.35)	1,060 (1.89)	986 (2.31)	509 (2.80)	275 (4.06)	138 (5.44)	
	Current	119,551 (94.83)	53,184 (95.00)	40,489 (95.06)	17,243 (94.85)	6,321 (93.38)	2,314 (91.21)	
Ethnic background (%)	European	121,934 (96.72)	54,384 (97.14)	41,279 (96.91)	17,439 (95.93)	6,426 (94.93)	2,406 (94.84)	<0.001
	European or Asian or African mixed race	634 (0.50)	175 (0.31)	205 (0.48)	130 (0.72)	83 (1.23)	41 (1.62)	
	Asian	1,185 (0.94)	552 (0.99)	359 (0.84)	183 (1.01)	65 (0.96)	26 (1.02)	
	African	953 (0.76)	349 (0.62)	291 (0.68)	191 (1.05)	92 (1.36)	30 (1.18)	
	Others	1,358 (1.08)	524 (0.94)	461 (1.08)	236 (1.30)	103 (1.52)	34 (1.34)	
IPAQ activity group (%)	Low	19,376 (17.93)	8,526 (17.85)	6,525 (17.83)	2,880 (18.34)	1,025 (17.52)	420 (19.29)	<0.001
	Moderate	46,529 (43.05)	20,943 (43.84)	15,797 (43.18)	6,569 (41.83)	2,382 (40.72)	838 (38.49)	
	High	42,187 (39.03)	18,304 (38.31)	14,266 (38.99)	6,255 (39.83)	2,443 (41.76)	919 (42.21)	
BMI (%)	≤ 18.5	710 (0.56)	372 (0.67)	207 (0.49)	86 (0.47)	32 (0.47)	13 (0.51)	<0.001
	18.5–24.9	49,057 (39.01)	23,310 (41.72)	16,004 (37.66)	6,632 (36.58)	2,315 (34.29)	796 (31.49)	
	25–29.9	51,951 (41.31)	22,926 (41.03)	17,821 (41.94)	7,472 (41.21)	2,759 (40.86)	973 (38.49)	
	≥ 30	24,053 (19.10)	9,262 (16.56)	8,459 (19.89)	3,940 (21.51)	1,646 (24.36)	746 (29.49)	
TDI (Mean ± SD)		−1.759 (2.801)	−1.960 (2.688)	−1.767 (2.785)	−1.484 (2.920)	−1.167 (3.084)	−0.724 (3.240)	<0.001
Education (%)	University	17,384 (13.82)	7,896 (14.14)	5,819 (13.69)	2,441 (13.45)	906 (13.42)	322 (12.72)	<0.001
	Below university or other professional qualifications	100,235 (79.68)	44,643 (79.92)	33,775 (79.44)	14,481 (79.80)	5,341 (79.14)	1995 (78.79)	
	None of the above	8,184 (6.51)	3,322 (5.95)	2,921 (6.87)	1,224 (6.75)	502 (7.44)	215 (8.49)	
Depression (%)	No	111,068 (88.10)	50,765 (90.68)	37,589 (88.25)	15,314 (84.24)	5,488 (81.08)	1912 (75.36)	<0.001
	Yes	14,996 (11.90)	5,219 (9.32)	5,006 (11.75)	2,865 (15.76)	1,281 (18.92)	625 (24.64)	
Anxiety (%)	No	116,094 (92.09)	51,906 (92.72)	39,165 (91.95)	16,606 (91.35)	6,139 (90.69)	2,278 (89.79)	<0.001
	Yes	9,970 (7.91)	4,078 (7.28)	3,430 (8.05)	1,573 (8.65)	630 (9.31)	259 (10.21)	
Anxiety-depression co-morbidity (%)	No	118,702 (94.16)	53,808 (96.11)	40,300 (94.61)	16,602 (91.33)	5,964 (88.11)	2028 (79.94)	<0.001
	Yes	7,362 (5.84)	2,176 (3.89)	2,295 (5.39)	1,577 (8.67)	805 (11.89)	509 (20.06)	
At least one (%)	No	93,736 (74.36)	44,511 (79.51)	31,864 (74.81)	12,164 (66.91)	4,053 (59.88)	1,144 (45.09)	<0.001
	Yes	32,328 (25.64)	11,473 (20.49)	10,731 (25.19)	6,015 (33.09)	2,716 (40.12)	1,393 (54.91)	

ACEs, adverse childhood experiences; IPAQ, International Physical Activity Questionnaire; BMI, body mass index; TDI, Townsend Deprivation Index.

### 3.2 ACEs with psychiatric disorders risk in the full cohort

As shown in Table 2, a positive correlation was found between the ACEs score and outcome variables. When ACEs score ≥ 4, the outcome variables in descending order of psychiatric disorders risk were anxiety-depression co-morbidity (OR = 4.87, 95% CI: 4.37 ~ 5.43), at least one (OR = 3.90, 95% CI: 3.59 ~ 4.23), depression (OR = 2.54, 95% CI: 2.31 ~ 2.80) and anxiety (OR = 1.38, 95% CI: 1.21 ~ 1.58). Smoking status, alcohol consumption, and TDI (except for anxiety) were all positively correlated with psychiatric disorders, while IPAQ physical activity was negatively correlated with psychiatric disorders. Compared with other participants in the same group, obesity (BMI ≥ 30) had a higher correlation with depression (OR = 1.75, 95% CI: 1.37–2.26) and at least one (OR = 0.21, 95% CI: 1.02–1.43). Meanwhile, when an individual has a below college degree, there is a higher correlation with anxiety (OR = 1.13, 95% CI: 1.06–1.20), anxiety-depression co-morbidity (OR = 1.09, 95% CI: 1.01–1.18), and at least one (OR = 1.06, 95% CI: 1.02–1.10).

**Table 2:** Logistic regression models for the relationship between ACE scores and outcomes.

Variables	Depression OR (95% CI)	Anxiety OR (95% CI)	Anxiety-depression co-morbidity OR (95% CI)	At least one OR (95% CI)
ACE Score	Reference	Reference	Reference	Reference
Score 1	1.26 (1.21, 1.31) ***	1.12 (1.07, 1.17) ***	1.36 (1.28, 1.45) ***	1.28 (1.24, 1.32) ***
Score 2	1.69 (1.61, 1.78) ***	1.21 (1.14, 1.28) ***	2.16 (2.02, 2.32) ***	1.82 (1.75, 1.88) ***
Score 3	1.98 (1.85, 2.12) ***	1.29 (1.18, 1.41) ***	2.86 (2.62, 3.12) ***	2.32 (2.20, 2.45) ***
Score ≥ 4	2.54 (2.31, 2.80) ***	1.38 (1.21, 1.58) ***	4.87 (4.37, 5.43) ***	3.90 (3.59, 4.23) ***
Age	0.99 (0.98, 0.99) ***	1.01 (1.01, 1.02) ***	0.98 (0.97, 0.98) ***	0.99 (0.98, 0.99) ***
Sex				
Female	Reference	Reference	Reference	Reference
Male	0.57 (0.55, 0.59) ***	0.66 (0.63, 0.69) ***	0.58 (0.55, 0.62) ***	0.53 (0.52, 0.55) ***
Smoking status				
Never	Reference	Reference	Reference	Reference
Previous	1.23 (1.18, 1.27) ***	1.16 (1.11, 1.20) ***	1.27 (1.21, 1.34) ***	1.28 (1.24, 1.31) ***
Current	1.53 (1.43, 1.62) ***	1.08 (0.99, 1.72)	1.54 (1.42, 1.68) ***	1.53 (1.46, 1.61) ***
Alcohol drinker status				
Never	Reference	Reference	Reference	Reference
Previous	1.29 (1.12, 1.49) ***	1.32 (1.11, 1.58) *	1.66 (1.38, 2.00) ***	1.59 (1.42, 1.77) ***
Current	0.99 (1.89, 1.10)	1.08 (0.95, 1.23)	0.92 (0.80, 1.07)	1.00 (0.92, 1.08)
Ethnic background				
White	Reference	Reference	Reference	Reference
Mixed	0.92 (0.73, 1.15)	0.71 (0.51, 0.99) *	0.78 (0.57, 1.06)	0.78 (0.65, 0.93) **
China	0.77 (0.63, 0.94) *	0.76 (0.59, 0.98) *	0.48 (0.34, 1.67) ***	0.64 (0.55, 0.75) ***
Black	0.51 (0.40, 0.64) ***	0.70 (0.53, 0.93) *	0.46 (0.33, 0.63) ***	0.47 (0.40, 0.56) ***
Others	0.95 (0.81, 1.12)	0.79 (0.63, 0.99) *	0.69 (0.54, 0.89) **	0.80 (0.70, 0.91) **
IPAQ activity group				
Low	Reference	Reference	Reference	Reference
Moderate	0.95 (0.90, 1.00) *	0.97 (0.91, 1.03)	0.87 (0.81, 0.93) ***	0.92 (0.88, 0.96) ***
High	0.89 (0.84, 0.94) ***	0.93 (0.88, 0.99) *	0.82 (0.77, 0.88) ***	0.86 (0.83, 0.90) ***
Education				
University	Reference	Reference	Reference	Reference
Below university or other professional qualifications	0.98 (0.93, 1.03)	1.13 (1.06, 1.20) **	1.09 (1.01, 1.18) **	1.06 (1.02, 1.10) **
None of the above	0.93 (0.86, 1.02)	1.11 (1.00, 1.22) *	1.11 (0.99, 1.24)	1.03 (0.97, 1.10)
BMI				
≤ 18.5	Reference	Reference	Reference	Reference
18.5–24.9	1.24 (0.97, 1.60)	1.04 (0.80, 1.36)	0.61 (0.47, 0.80) **	0.96 (0.81, 1.14)
25–29.9	1.39 (1.08, 1.78) *	0.99 (0.76, 1.30)	0.68 (0.53, 0.89) **	1.03 (0.87, 1.22)
≥ 30	1.75 (1.37, 2.26) ***	0.94 (0.72, 1.23)	0.77 (0.59, 1.00)	1.21 (1.02, 1.43) *
TDI	1.02 (1.02, 1.03) ***	1.00 (1.00, 1.01)	1.03 (1.02, 1.04) ***	1.03 (1.02, 1.03) ***

It was adjusted for age, sex, smoking status, alcohol drinker status, ethnic background, IPAQ activity group, education, BMI, and TDI among 126,064 participants (full cohort). ACEs, adverse childhood experiences; IPAQ, International Physical Activity Questionnaire; BMI, body mass index; TDI, Townsend Deprivation Index; boldface indicates statistical significance (*0.01 < *p* value < 0.05, **0.001 < *p* value < 0.01, ***p value < 0.001).

### 3.3 Prevalence of ACEs in different groups

**Figure 2:**
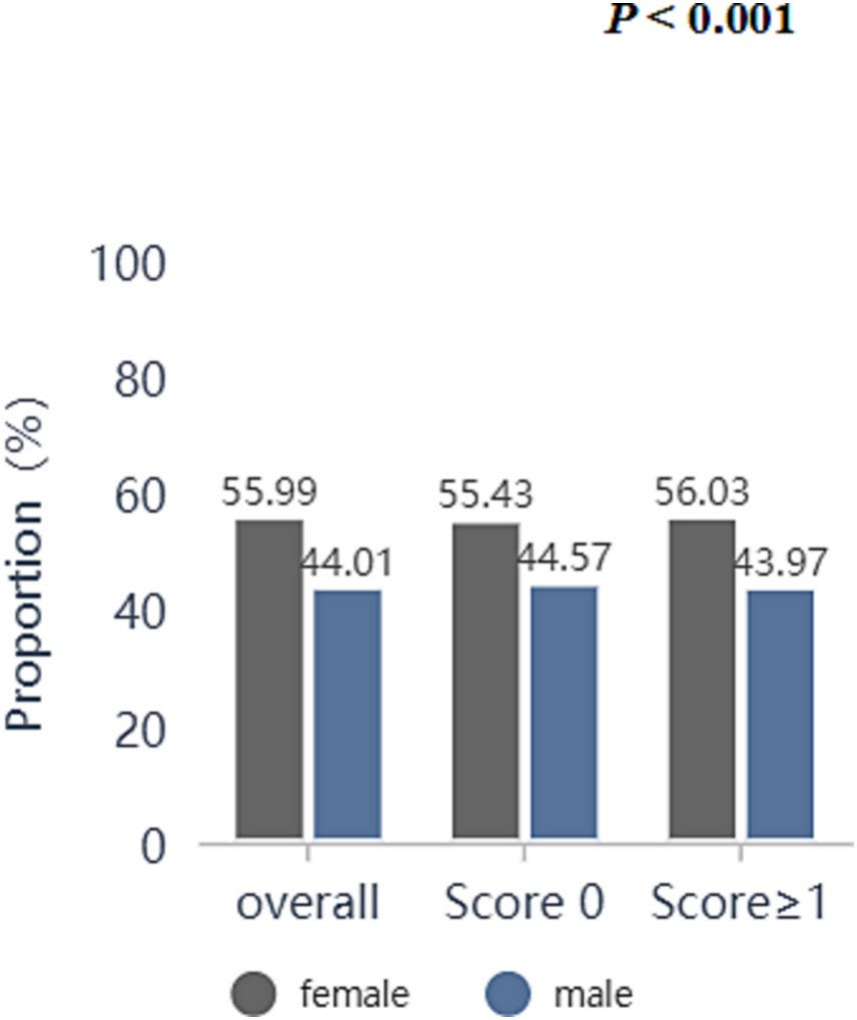
Prevalence of ACEs stratified by sex in the 2006–2022 UK Biobank.

Overall, there was a significant difference in prevalence between males and females in the different subgroups of ACEs (*p* < 0.001). 44.01% of males and 55.99% of females participated in the study (Figure 2). The prevalence of ACEs in the female (56.03%) group was higher than that in the male (43.97%) group (*p* < 0.001) (Figure 2).

### 3.4 ACEs with psychiatric disorders risk at different sex in full and European cohort

**Figure 3:**
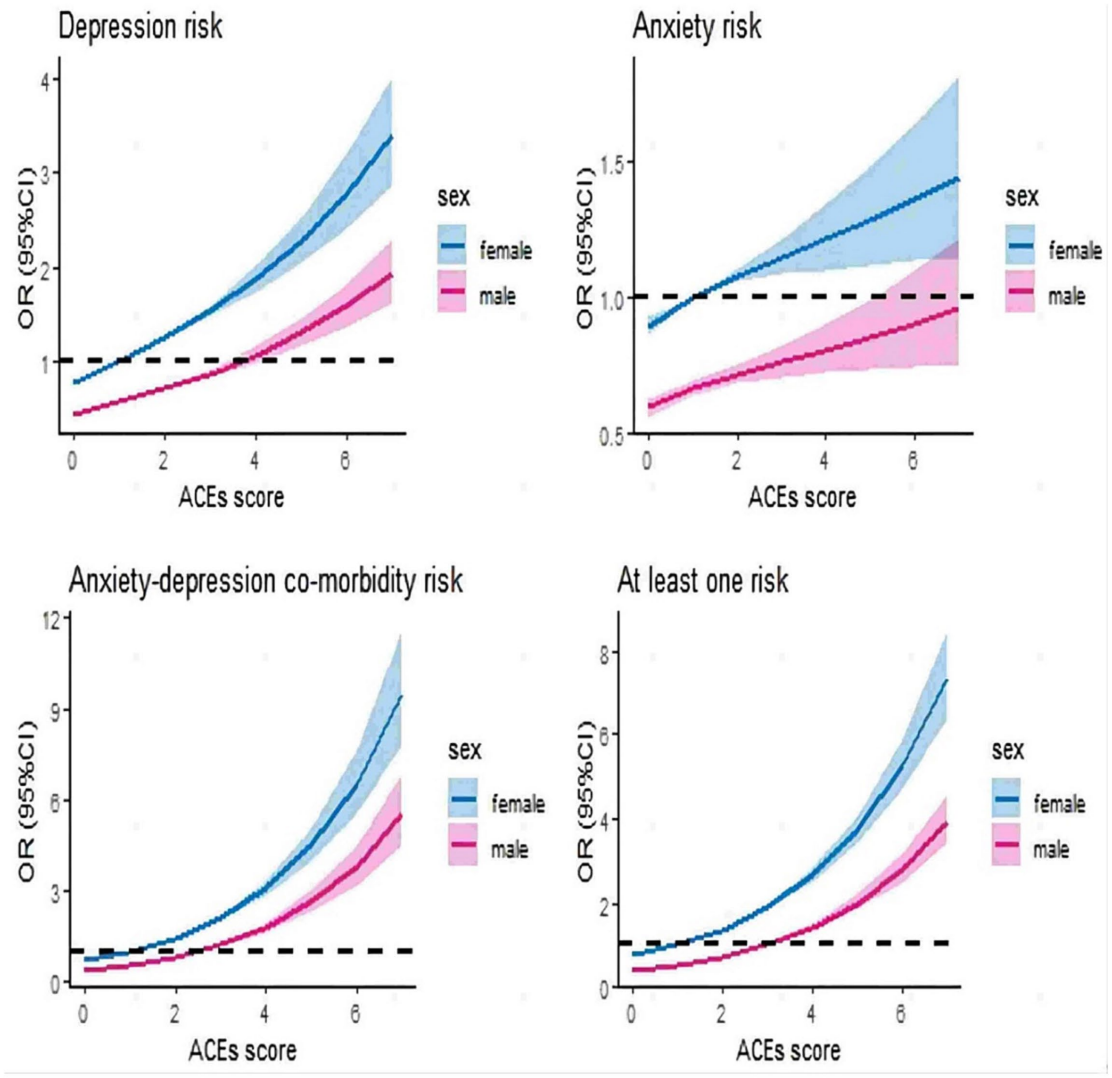
Restricted cubic spline models for relationship between ACEs and outcomes at different sex groups (full cohort). It was adjusted for age, sex, smoking status, alcohol drinker status, ethnic background, IPAQ activity group, education, BMI and TDI among 126,064 participants (full cohort), grouped according to sex.

Figure 3 shows results from the restricted cubic spline models for the relationship between categories of ACE and outcome variables in the full cohort, grouped by sex and controlling for confounders. In several groups, there was a dose-dependent relationship between ACEs and outcome variables (*p* < 0.05 for non-linear tests of depression and at least one, *p* > 0.05 for non-linear tests of anxiety and anxiety-depression co-morbidity) and higher for females than males. Results for females showed that ACE = 0 was linked with the lowest odds ratio of incident depression (OR = 0.77, 95% CI: 0.74 ~ 0.79), anxiety (OR = 0.90, 95% CI: 0.86 ~ 0.93), anxiety-depression co-morbidity (OR = 0.70, 95% CI: 0.67 ~ 0.73) and at least one (OR = 0.77, 95% CI: 0.74 ~ 0.80). The risk of depression (OR = 0.88, 95% CI: 0.83 ~ 0.93) in males was relatively flat at ACEs = 0–3, while the risk of anxiety-depression co-morbidity (OR = 0.85, 95% CI: 0.80 ~ 0.90) and at least one (OR = 0.73, 95% CI: 0.70 ~ 0.75) at ACEs = 0–3 relatively flat, followed by a gradual increase. Hereafter, the risk ratios for anxiety in males were all less than 1, with the highest result being 0.96 (95% CI: 0.76 ~ 1.26) at ACEs = 7. Besides, females (OR = 9.52, 95% CI: 7.81 ~ 11.61) and males (OR = 5.57, 95% CI: 4.53 ~ 6.86) with ACEs score of 7 has the highest risk ratio for anxiety-depression co-morbidity. Comparative results by sex in the European cohort and by age in the full cohort are shown in Supplementary Figures S1, S2.

### 3.5 Category of ACEs with psychiatric disorders risk in the full cohort

**Figure 4:**
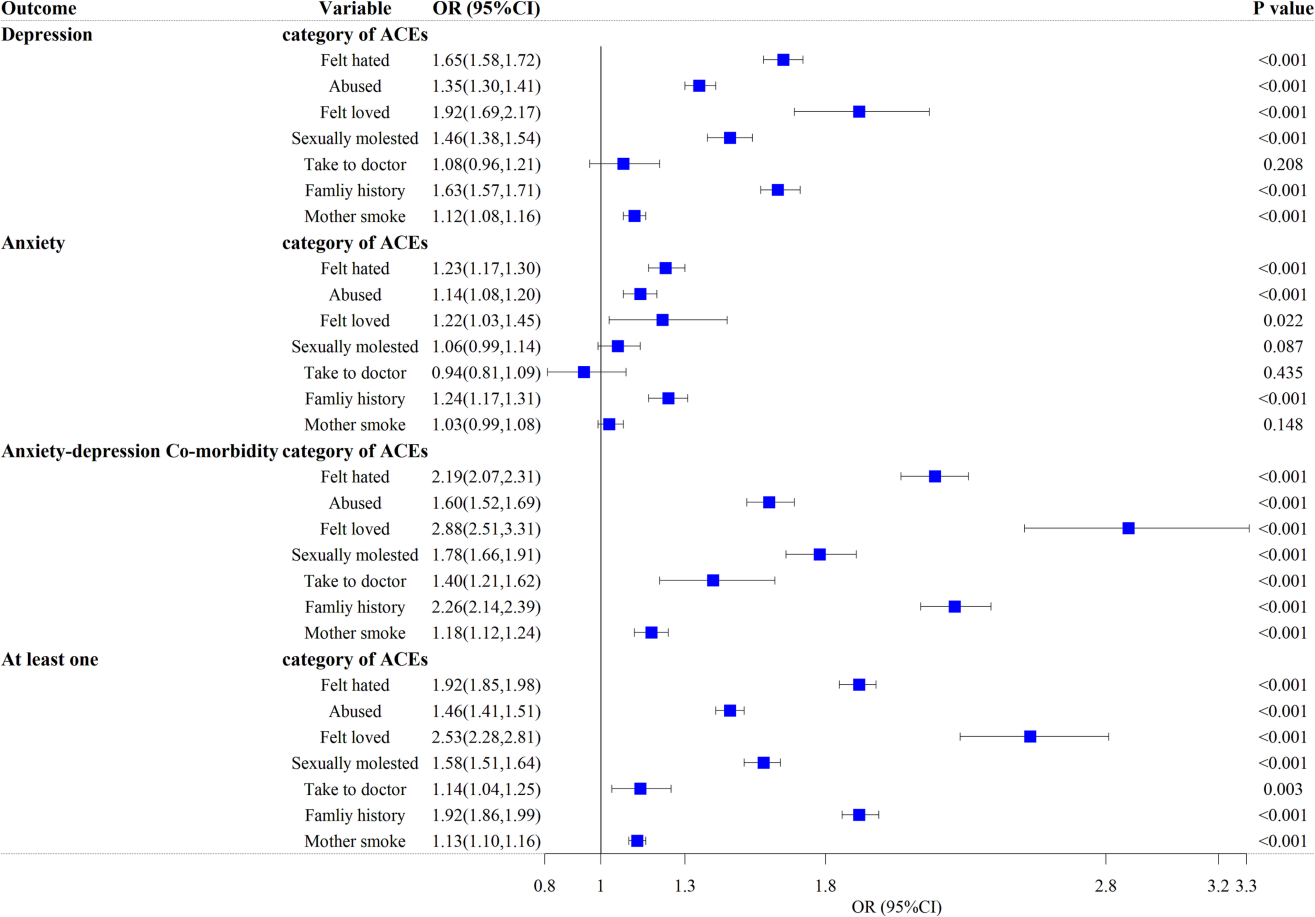
Logistic regression model for the relationship between individual categories of ACE and outcomes. It was adjusted for age, sex, smoking status, alcohol drinker status, ethnic background, IPAQ activity group, education, BMI and TDI among 126,064 participants (full cohort). Felt hated, felt hated by family member as a child; Abused, physically abused by family as a child; Felt loved, felt loved as a child; Sexually molested, sexually molested as a child; Take to doctor, someone to take to doctor when needed as a child; Family history, having a family history of psychosis; Mother smoke, maternal smoking around birth.

Figure 4 shows the logistic regression results between the category of ACEs and the outcome variables, also controlling for confounders. Overall, most of the results were significant, but the correlation between being taken to the doctor when needed as a child and depression or anxiety, the correlation between maternal smoking around birth and anxiety, and the correlation between being sexually molested as a child and anxiety were not significant (*p* > 0.05). It was found that the rates of anxiety-depression co-morbidity were always the highest. Compared to other categories of ACEs, in the OR values of all diseases (in addition to anxiety), felt loved as a child (reverse rating) was the highest value, in the following order (from left to right in Figure 4), OR = 1.92 (95% CI: 1.69 ~ 2.17), OR = 2.88 (95% CI: 2.51 ~ 1.31), OR = 2.53 (95% CI: 2.28 ~ 2.81). Those with a family history of psychiatric disorders (OR = 1.24, 95% CI: 1.17 ~ 1.31) had the greatest values when suffering from anxiety. Feeling hated by family members as a child was associated with depression (OR = 1.65, 95% CI: 1.58 ~ 1.72), anxiety (OR = 1.23, 95% CI: 1.17 ~ 1.30), anxiety-depression co-morbidity (OR = 2.19, 95% CI: 2.07 ~ 2.31) and at least one (OR = 1.92, 95% CI: 1.85 ~ 1.98) increased probability showed a positive correlation. Physically abused by family as a child was associated with depression (OR = 1.35, 95% CI: 1.30 ~ 1.41), anxiety (OR = 1.14, 95% CI: 1.08 ~ 1.20), anxiety-depression co-morbidity (OR = 1.60, 95% CI: 1.52 ~ 1.69) and at least one (OR = 1.46, 95% CI: 1.41 ~ 1.51) were significantly associated with increased odds of prevalence. Sexually molested as a child was significantly associated with depression (OR = 1.46, 95% CI: 1.38 ~ 1.54), anxiety-depression co-morbidity (OR = 1.78, 95% CI: 1.66 ~ 1.91), and at least one (OR = 1.58, 95% CI: 1.51 ~ 1.64). An increased odds for someone to take to the doctor when needed as a child (reverse rating) was associated with anxiety-depression co-morbidity (OR = 1.78, 95% CI: 1.66 ~ 1.91) and at least one (OR = 1.58, 95% CI: 1.51 ~ 1.64). Having a family history of psychosis was associated with increased odds of having depression (OR = 1.63, 95% CI: 1.57 ~ 1.71), anxiety-depression co-morbidity (OR = 2.26, 95% CI: 2.14 ~ 2.39), and at least one (OR = 1.92, 95% CI: 1.86 ~ 1.99). Maternal smoking at birth was significantly associated with depression (OR = 1.12, 95% CI: 1.08 ~ 1.16), anxiety-depression co-morbidity (OR = 1.18, 95% CI: 1.12 ~ 1.24), and at least one (OR = 1.13, 95% CI: 1.10 ~ 1.16).

## 4 Discussion

This study used a large, diverse, and multicultural dataset from the UK Biobank to discover the dose–response relationship between ACE, depression, anxiety, at least one, and comorbidity of anxiety and depression through our research, thus validating the previous research some findings in the relevant literature. Considering the difference in race and age, we further verified the above relationship, and the final result showed that the prevalence of anxiety-depression co-morbidity was the highest. We also suggested significant differences between categories of ACEs and anxiety-depression co-morbidity, controlling for relevant demographic and socioeconomic factors.

In this study, 55.59% reported at least one ACE, which is within the range reported in the literature (46.4–79.5%) (3). As expected from our first hypothesis, in some literature (21, 22, 26) ACEs increase the probability of adult exposure to psychiatric disorders and risky behaviors. In the present study, ACEs were highest when anxiety-depression co-morbidity was present. Although the number of anxiety-depression co-morbidity is less than depression, it had the highest value in the results because it had both anxiety and depression symptoms compared to a single psychiatric disorder. Another point mentioned is the research on the correlation between ACEs and psychiatric disorders, female, smoking history, low education, drinking history, overweight or obesity, and TDI are all risk factors; frequent physical activity is a protective factor. The above results are the same as those of this literature (38–40). As early ACE-induced mood changes trigger a biological stress response, it leads to an impact on the HPA axis, stimulating the adrenal cortex to secrete cortisol at persistently high levels for a prolonged period, placing the individual at an increased risk of developing depression and anxiety disorders (24). The higher risk of females in this compared to males may be due to the fact that females themselves have higher cortisol levels than males; both smoking and drinking are the results of compensation for bad childhood behaviors (41). TDI indices tend to reflect socio-economic levels at the regional level (42), with higher scores indicating poorer areas (35), which may put pressure on parents to make children more vulnerable to ACEs. Previous research has shown that social background is an important factor influencing ACEs (43). For example, in a study based on a representative sample of the German population, participants from West Germany/foreign countries were at a higher risk of experiencing ACEs compared to East Germany, where state-directed child care is available (44). Ethnicity may be limited by the area of data collection, with Europeans having a higher prevalence in comparison to other ethnicities.

As expected from our second hypothesis, our results show that the dose-dependent relationship between ACEs and psychiatric disorders differed between participants of different sex and ages. We also selected the European group due to the largely white population. In addition to cortisol, Robert C. Whitaker’s study of the interaction between ACEs and depression or anxiety disorders and sex in U.S. adults suggests that the synergistic effect of ACEs and females on anxiety or depression is greater than the separate effects of these two factors (26).

As expected from our third hypothesis, our results showed significant differences between the category of ACEs and anxiety-depression co-morbidity. Feeling loved in childhood (this reverse score belongs to emotional neglect) was highest in depression and anxiety-depression co-morbidity. Family history of psychiatric disorders was highest in anxiety disorders. However, there were also nonsignificant categories, which suggest that the mechanisms of expression of each ACE may have different implications for a single psychiatric disorder. Mechanisms under a single expression: different types of abuse can reactively alter the HPA, thus impairing the emergency attachment system and leading to varying degrees of mood disturbance and increased or decreased cortisol concentrations (41). The lack of significance mentioned above may also be due to the significant difference between the number of patients and non-patients, resulting in low statistical power. It is worth noting that since at the outset, our assumptions for the definition of at least one was any of depression, anxiety, and anxiety-depression co-morbidity, taking into account the number of people with the condition became larger, the risk of overlap between the disorders (45) (the shared risk of the two disorders, not their co-morbidities) increased, and the effect of unmeasured factors on the results. There may be similarities between the anxiety-depression co-morbidity and its coefficients. Interestingly, the coefficient of anxiety-depression co-morbidity was the highest because of the coexistence of depressive and anxiety symptoms compared to other disorders, although the number was relatively small.

The strength of this study design was that based on a well-established large cohort from Europe, controlled the confounding factors related to ACE and psychiatric disorders, studied the correlation between each category of ACEs or scores and the comorbidity of common psychological diseases, and most of the results are significant. This suggests that the public should be concerned not only about the risky behaviors (smoking, drinking, etc.) and individual diseases (hypertension, depression, etc.) caused by ACEs in adulthood, but also about the harms caused by the anxiety-depression co-morbidity. Of course, this study also has limitations. First, with respect to disease, self-reported non-cancer diseases were selected in our study section to determine prevalence, which may introduce recall and measurement bias, and the order of diseases after recruitment has not been considered; Second, in terms of confounders, chronic diseases, and genetic factors were not considered; Third, in terms of the independent variable, the ACE scale is not absolutely suitable for European, we did not consider the prevalence of different ACEs combinations and individual ACEs are not graded.

## 5 Conclusion

In conclusion, although there are many previous studies on the relationship between single psychiatric disorders and ACEs, we should be aware that the potential impact of comorbidity cannot be ignored. The present study showed that with an increase in the number of ACEs or the manifestation of a single ACE, participants had a higher probability of anxiety-depression comorbidity. Therefore, only early intervention of adverse life factors, protection of the emergency attachment system, and control of cortisol hormones can prevent and control public mental health and improve the quality of life.

## Data availability statement

Publicly available datasets were analyzed in this study. This data can be found here: this study used the UK Biobank resource with the application ID 88159. Researchers can access the UK Biobank by applying to the UK Biobank official website (https://www.ukbiobank.ac.uk/).

## Ethics statement

The UK Biobank database has been approved by the Research Tissue Bank (RTB) with the North West Multicentre Research Ethics Committee (MREC) consent, meaning that each participant does not have to sign a separate consent form and proceeds directly under the approval of the RTB.

## Author contributions

KW, YJ, and LH put forward the idea of this research. PZ obtained data. ZJ and PY clean up the dataset and analyze the data. PY and CZ explained the results of data analysis and wrote a manuscript. All authors contributed to the article and approved the submitted version.

## Footnotes

^1^www.ukbiobank.ac.uk/resources/

^2^https://biobank.ctsu.ox.ac.uk/crystal/field.cgi?id=41270
