# Supplementary material for: Variety ACEs and risk of developing anxiety, depression, or anxiety-depression co-morbidity: the 2006–2022 UK Biobank data
Source: Front Psychiatry. 2023 Dec 27;14:1233981. doi: 10.3389/fpsyt.2023.1233981 (PMC10793109; doi:10.3389/fpsyt.2023.1233981)
Supplement: Supplementary file 1 [file Data_Sheet_1.docx]

Supplementary Material

Variety ACEs and risk of developing anxiety, depression, or anxiety-depression co-morbidity: the 2006-2022 UK Biobank data

# Peilin Yu^1^, Zhou Jiang^1^, Chu Zheng^1,2,3,4^, Ping Zeng^1,2,3,4^, Lihong Huang^5*^, Yingliang Jin^1,2,3,4*^, Ke Wang^1,2,3,4*^

# * Correspondence: Corresponding Author:

# Ke Wang, E-mail:[xzmuwk@xzhmu.edu.cn](mailto:xzmuwk@xzhmu.edu.cn)

# 1 Supplementary Figures and Tables

# 1.1 Supplementary Figures


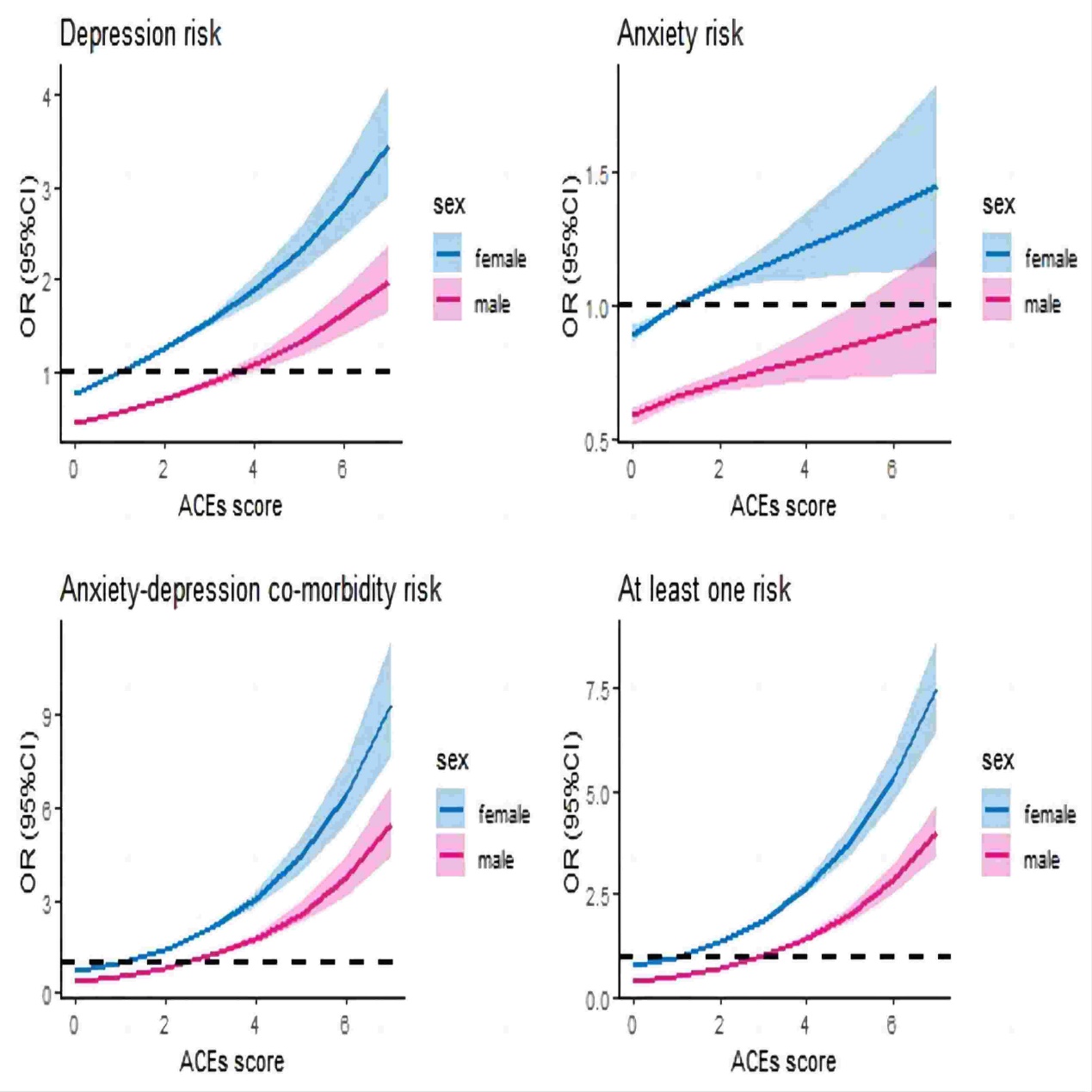


**Supplementary Figure 1** Restricted cubic spline models for the relationship between ACEs and outcomes at different sex groups (European cohort).

**Notes:** It was adjusted for age, smoking status, alcohol drinker status, ethnic background, IPAQ group, education, BMI, and TDI among 121934 participants (European cohort), at different sex groups.


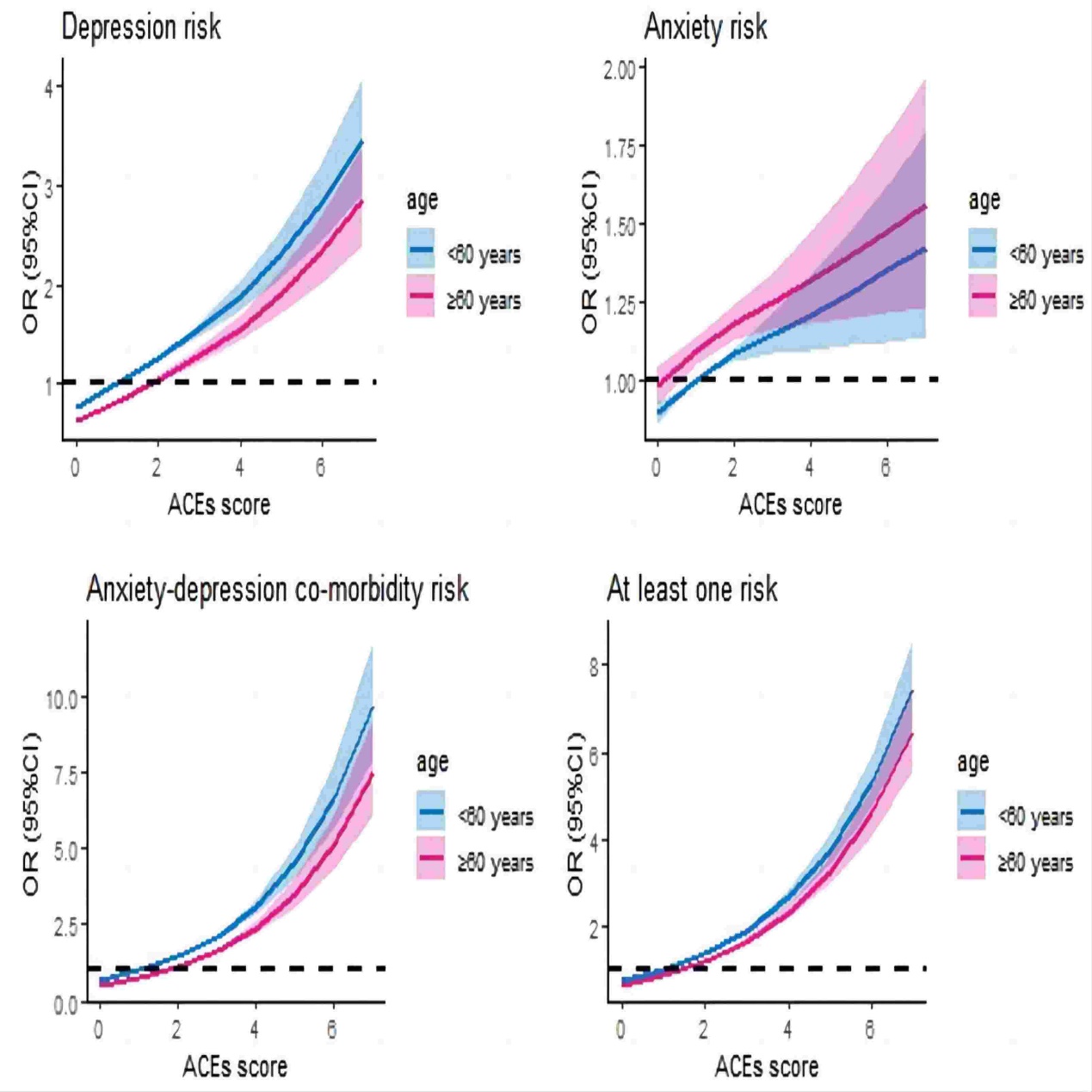


**Supplementary Figure 2** Restricted cubic spline models for the relationship between ACEs and outcomes at different age groups (full cohort).

**Notes:** It was adjusted for sex, smoking status, alcohol drinker status, ethnic background, IPAQ group, education, BMI, and TDI among 126064 participants (full cohort), at different age groups.

# 1.2 Supplementary Tables

**Supplementary Table 1** Description of the category of adverse childhood experiences

|  | **Coding** | **ACEs Categories** |
| --- | --- | --- |
| Felt hated by a family member as a child | 20487 | Emotional abuse |
| Physically abused by family as a child | 20488 | Domestic violence |
| Felt loved as a child | 20489 | Emotional neglect |
| Sexually molested as a child | 20490 | Sexual abuse |
| Someone to take to the doctor when needed as a child | 20491 | Physical neglect |
| Maternal smoking around birth | 1787 | Household substance abuse |
| Illnesses of father | 20107 | Mental illness in the household |
| Illnesses of mother | 20110 |  |
| Illnesses of siblings | 20111 |  |

**Abbreviations:** ACEs, Adverse childhood experiences

**Supplementary Table 2** Definitions of the psychiatric disorders

| **Disease** | **After baseline** |
| --- | --- |
| Depression | 20002: 1286,1288,1289,1290,1291  ICD-10: F31, F32, F33 |
| Anxious | 20002: 1287  ICD-10: F40, F41, F42, F43, F44, F45, F48 |

**Supplementary Table 3** Description of the missing data

|  | **Missing values** |  | **Percentage of missingness (%)** |
| --- | --- | --- | --- |
| Age (years) | 0 |  | 0 |
| Sex (%) | 0 |  | 0 |
| Smoking status (%) | 0 |  | 0 |
| Alcohol drinker status (%) | 0 |  | 0 |
| Ethnicity (%) | 0 |  | 0 |
| IPAQ activity group (%) | 17,972 |  | 0.143 |
| BMI (%) | 293 |  | 0.002 |
| TDI | 154 |  | 0.001 |
| Education (%) | 261 |  | 0.002 |

**Abbreviations:** BMI, body mass index; IPAQ, International Physical Activity Questionnaire activity group; TDI, Townsend Deprivation Index
